# Learnings from clinical trials in patients with connective tissue disease-associated interstitial lung disease

**DOI:** 10.1186/s13075-023-03090-y

**Published:** 2023-07-08

**Authors:** Jean Paul Higuero Sevilla, Areeka Memon, Monique Hinchcliff

**Affiliations:** 1 Department of Internal Medicine, Section of Pulmonary, Critical Care and Sleep Medicine, Yale School of Medicine, New Haven, CT 06520 USA; 2 Department of Internal Medicine, University of Connecticut School of Medicine, Farmington, CT 06032 USA; 3 Department of Internal Medicine, Section of Rheumatology, Allergy & Immunology, Yale School of Medicine, 300 Cedar Street, The Anlyan Center PO BOX 208031, New Haven, CT 06520 USA

**Keywords:** Scleroderma, Systemic sclerosis, Interstitial lung disease, Inflammatory lung disease, Fibrotic lung disease, Connective tissue disease, Nintedanib, Pirfenidone, Tocilizumab, Rituximab

## Abstract

Many clinical trial results are available to inform best practices in the treatment of patients with connective tissue disease-associated interstitial lung disease (CTD-ILD).

Herein, we summarize the results of clinical trials, including patient-reported outcome instruments, for the treatment of patients with ILD associated with systemic sclerosis (SSc/scleroderma), rheumatoid arthritis, and idiopathic inflammatory myositis, the diseases with the most available data. For SSc-ILD, the US Food and Drug Administration approved nintedanib (a tyrosine kinase inhibitor) in 2020 and subcutaneous tocilizumab (an IL-6 receptor monoclonal antibody) in 2021. Rituximab was recently shown to have similar efficacy but better tolerability than intravenous cyclophosphamide (CYC) for CTD-ILD therapy. Scleroderma Lung Study II, conducted in patients with SSc-ILD, showed that oral CYC and mycophenolate mofetil (MMF) were comparable in their effects on lung function, but MMF was better tolerated. The increasing treatment armamentarium for patients with CTD-ILD offers physicians new opportunities to improve patient outcomes.

## Background

Pulmonary involvement, particularly interstitial lung disease (ILD), is a significant cause of morbidity and mortality in patients with connective tissue diseases (CTDs). The risk of developing ILD varies widely among CTDs (Table 1), but its presence is associated with worse prognosis [1]. There is significant variability in clinical presentation ranging from an asymptomatic incidental radiographic finding to rapidly progressive respiratory failure. ILD is sometimes identified in the setting of a known CTD. Still, other times, it can be the initial manifestation of an occult CTD requiring additional evaluation for diagnosis. The imaging on chest high-resolution computed tomography (HRCT) (Table 2) and histopathologic findings of CTD-associated ILD (CTD-ILD) may resemble those seen in idiopathic ILDs. The usual diagnostic approach requires a thorough evaluation looking for CTD signs and symptoms and consideration of alternative etiologies such as environmental exposures and drug toxicity. Multidisciplinary case discussions increase diagnostic consensus and confidence and are endorsed in guidelines [2].

**Table 1:** Frequency of interstitial lung disease in some connective tissue diseases

Disease	ILD prevalence	Comment
Systemic sclerosis	Up to 80% (on imaging) 30–40% (clinical disease) [3]	+ anti-Scl-70 is a risk factor
Polymyositis/dermatomyositis	Up to 65–78% (imaging and PFTs) [4]	More common with anti-synthetase antibodies
Rheumatoid arthritis	1–73% [5]	Rare in seronegative disease
Systemic lupus erythematosus	3–9% [6]	
Sjogren’s syndrome	10–20% [7]

**Table 2:** Chest high-resolution computed tomography (HRCT) patterns in CTD-ILD

Disease	CT pattern
Systemic sclerosis	NSIP > UIP > O > OP
Myositis	NSIP = UIP > OP
Rheumatoid arthritis	UIP > NSIP > OP
Systemic lupus erythematosus	OP > NSIP > UIP
Sjogren’s syndrome	NSIP > UIP > OP > LIP

Adapted from [8, 9]

*LIP* lymphoid interstitial pneumonia, *NSIP* non-specific interstitial pneumonia, *O* other, *OP* organizing pneumonia, *UIP* usual interstitial pneumonia

The treatment of ILD has focused on reducing inflammation and/or preventing progression of lung function decline, thereby improving survival or how a patient feels and/or functions (Table 3). Idiopathic pulmonary fibrosis (IPF) has the most evidence to guide therapy. Antifibrotic therapy showed benefit in slowing progression of IPF as assessed by pulmonary function tests (PFTs) [10, 11]. The combination of prednisone and azathioprine was shown to be harmful and associated with increased mortality [12]. The decision to start or escalate CTD-ILD therapy should involve shared medical decision-making with the patient, and be informed by disease severity, evidence or likelihood of progression, and extra-thoracic manifestations. Historically, CTD-ILD treatment was based on expert opinion due a paucity of randomized controlled trial (RCT) data. Now, a host of clinical trial data are available to inform therapeutic decisions, and these will be summarized herein.

**Table 3:** Common patient-reported outcome instruments used in CTD clinical trials

St. George’s Respiratory Questionnaire
Mahler Baseline Dyspnea Index (BDI) and Transition Dyspnea Index (TDI)
Functional Assessment of Chronic Illness Therapy (FACIT)-Dyspnea
Health Assessment Questionnaire-Disability Index (HAQ-DI)
King’s Brief Interstitial Lung Disease Questionnaire (KBILD)
Health Assessment Questionnaire-Disability Index (HAQ-DI)

## Connective tissue disease-specific immunosuppressive treatment

Results of the RECITAL trial, a 24-week, phase 2b double-blind randomized controlled trial of rituximab [(RTX), a CD20 receptor monoclonal antibody, 1000 mg IV at weeks 0 and 2, *n* = 51] vs. cyclophosphamide [(CYC), a nucleic acid alkylating agent, 600 mg/m^2^ body surface area IV every 4 weeks for six doses, *n* = 50] in patients with CTD-ILD demonstrated that RTX was not superior to CYC in improving forced vital capacity % predicted (FVC) [13]. Participants had severe or progressive (in the opinion of the treating physician) SSc (*n* = 37), idiopathic inflammatory myositis (*n* = 44), or mixed CTD (*n* = 16) with a HRCT demonstrating ILD within the preceding 12 months. Patients with prior RTX or CYC exposure were excluded, and no background or additional immunosuppression, other than oral glucocorticoids, were permitted until week 24 unless required clinically. The change in FVC between baseline and 24 weeks (primary endpoint) was 97 mL [standard deviation (SD) 234] in the RTX- and 99 mL (SD 329) in the CYC-treated group. In a mixed effects model adjusting for baseline FVC and CTD type, the difference in FVC at week 24 was − 40 mL in the RTX- vs. CYC-treated patients, favoring CYC, but the difference was not significant (*p* = 0.49). Secondary endpoints including change between baseline and week 48 in FVC; change between baseline and week 24 or 48 in 6-min walk distance, DL_CO_, quality of life scores on the St. George’s Respiratory Questionnaire (SGRQ), King’s Brief Interstitial Lung Disease (KBILD) questionnaire, and European Quality of Life Five-Dimension (EQ-5D) questionnaire; overall survival, progression-free survival, and time to treatment failure; and glucocorticoid (GC) use, were comparable between groups. The change in Physician Global Visual Analog Scale (VAS) between baseline and week 48 favored CYC [13]. In the RTX vs. CYC groups, there were fewer patients with serious adverse events (*n* = 29 vs. *n* = 33), less glucocorticoid exposure [11,469 mg (SD 10,041) vs. [13,239 mg (SD 14,657)], and less need for maintenance immunosuppression (azathioprine, methotrexate, mycophenolate mofetil, or tacrolimus) at week 24 (*n* = 33 vs. *n* = 39), rendering RTX a potential treatment for progressive CTD-ILD.

## Systemic sclerosis-associated ILD (SSc-ILD)

Systemic sclerosis is the CTD most frequently associated with ILD, with a reported prevalence of up to 80% of patients in imaging studies [3]. Male sex, older age, African-American race, diffuse skin disease, and positivity for anti-Scl-70 antibodies are reported risk factors for SSc-ILD development. It is the CTD for which the most data exist to guide therapy [14–16]. SSc-ILD is associated with significant morbidity and mortality, making early detection and treatment crucial [17].

Glucocorticoids (GC) were traditionally utilized as part of the treatment regimen for SSc-ILD based on experience and limited data [18]. Glucocorticoid dose was typically maintained below 15 mg of prednisone daily due to concern for the potential association between GC and scleroderma renal crisis. Subsequently, based on significant concerns for toxicity and the availability of alternative therapies with higher quality evidence, GC have fallen out of favor for SSc-ILD [19].

Scleroderma Lung Study I (SLS I) was a multicenter RCT where patients with SSc-ILD received oral CYC up to 2.0 mg/kg/day or placebo for 12 months followed by standard of care treatment for 12 months. One year of treatment resulted in a small benefit in lung function (mean difference in adjusted change from baseline in FVC % predicted at 12 months was 2.53% [95% CI 0.28–4.79%]) in favor of CYC) [16]. Health-related quality of life measures also improved in the active treatment group as assessed by the (Mahler) Baseline Dyspnea Index/Transition Dyspnea Index, Modified Cough Index, 36-Item Medical Outcomes Survey (SF-36), and 20-Item Scleroderma Health Assessment Questionnaire-Disability Index (SHAQ-DI). However, only dyspnea remained improved, while FVC % predicted improvement was lost, at 52 weeks post-treatment [20]. These data suggest that immune suppression beyond 12 months is needed for SSc-ILD treatment. Regarding patient-reported outcomes, the Transition Dyspnea Index score showed a clinically meaningful improvement in the treatment group (+ 1.4 ± 0.23) compared to clinically meaningful worsening in the placebo group (− 1.5 ± 0.43) at 12 months. The adjusted mean SHAQ-DI scores at 12 months were lower (better) in the treatment group compared to placebo (difference: − 0.16 [95% CI − 0.28 to − 0.04]).

Scleroderma Lung Study II (SLS II) randomized patients with SSc-ILD to CYC up to 2.3 mg/kg/day PO for 12 months followed by a placebo for 12 months, compared to mycophenolate mofetil [(MMF), inosine 5′-monophosphate dehydrogenase inhibitor] 1.5 g PO BID for 24 months. Lung function improved in both treatment arms (change in FVC % predicted at 2 years: 2.88 (95% CI 0.53–3.84) in CYC group vs. 2.19 (95% CI 1.19–4.58) in MMF group), but MMF was associated with fewer adverse events. Patient-reported outcomes included the Baseline Dyspnea Index/Transition Dyspnea Index (secondary outcome), Leicester Cough Questionnaire, SF-36, SGRQ, SHAQ-DI, Health Utilities, and UCLA Gastrointestinal Tract Instrument. Transition Dyspnea Index change between baseline and 24 months was 2.16 (95% CI 1.14–3.18) for CYC- and 1.77 (95% CI 0.75–2.79) for MMF-treated patients, indicating dyspnea improvement in both groups [14].

For SLS I, 42% of participants had died (CYC, *n* = 38 and placebo, *n* = 28) mostly of SSc-attributable deaths after a median follow-up of 8 years [21]. Interestingly, decline in FVC and DLCO over 2 years was a stronger predictor of death than either baseline FVC or DLCO. For SLS II, 21% of participants had died (CYC, *n* = 16 and MMF, *n* = 14) after a median follow-up of 3.6 years [21]. Fifteen (58%) deaths, among the 26 participants with known cause of death, were attributable to SSc, with respiratory failure underlying 13.

Due to CYC’s known toxicities, the Fibrosing Alveolitis in Scleroderma Trial (FAST) compared FVC % predicted change at 12 months between SSc patients with radiographic or histologic evidence of pulmonary fibrosis treated with active treatment (oral prednisolone 20 mg every other day and 6 monthly doses of IV CYC (600 mg/m^2^) followed by daily azathioprine (AZA, a purine metabolism antagonist) for an additional 6 months (*n* = 22) vs. placebo (*n* = 23). Although treatment was not associated with significant differences in FVC change between groups [22], CYC, but not AZA, was included in the 2017 European Alliance of Associations for Rheumatology (EULAR) recommendations as a therapy for progressive SSc-ILD [23].

The biologic disease-modifying anti-rheumatic drug (bDMARD) tocilizumab (monoclonal IL-6 receptor antibody) has been studied for the treatment of SSc skin disease and found, in secondary analyses, to reduce FVC decline in patients with ILD. In a phase II multicenter RCT (faSScinate), 87 patients with early dcSSc (≤ 5 years since first non-Raynaud sign or symptom) and modified Rodnan skin score 15–40 with evidence of active SSc (worsening skin thickening or ≥ 1 tendon friction rub plus elevated inflammatory markers [≥ 1 of C-reactive protein (CRP) ≥ 10 mg/L, erythrocyte sedimentation rate (ESR) ≥ 28 mm/h or platelets ≥ 330,000/μL]) were randomized to subcutaneous weekly tocilizumab 162 mg for 48 weeks versus placebo, followed by 48 weeks of open-label weekly tocilizumab. The study results demonstrated safety, but the primary endpoint, modified Rodnan skin score improvement, was not met. However, compared to placebo, fewer patients in the tocilizumab arm experienced an absolute decline in FVC % predicted > 10% at 48 weeks (10% vs. 23%). There was no significant difference at 48 weeks in the patient-reported outcomes [HAQ-DI, Patient Global VAS, Functional Assessment of Chronic Illness Therapy (FACIT)-Dyspnea score and 5D itch scale]. At baseline, patients in the tocilizumab group (mean FVC 80% predicted, mean DL_CO_ 73% predicted) and the placebo group (mean FVC 82% predicted, mean DL_CO_ 74% predicted) arms had mild lung function impairment [24].

A subsequent phase III multicenter RCT (focuSSced) of 210 patients with the same inclusion criteria as faSScinate was conducted. Patients were randomized to weekly subcutaneous tocilizumab 162 mg for 48 weeks vs. placebo (rescue immunosuppression was permitted at week 16). Again, the primary outcome of skin score change was not met. However, a post hoc subgroup analysis of SSc-ILD patients (*n* = 136) showed FVC stabilization with tocilizumab compared to placebo [change in FVC % predicted at week 48: 0.1 vs. − 6.4; difference 6.5 (95% CI: 3.4, 9.5), nominal *p* < 0.0001]. No significant between-group differences in patient-reported outcomes (SHAQ-DI VAS, Patient Global VAS, Physician Global VAS, or SGRQ) were noted between baseline and follow-up except for FACIT-Fatigue, which showed greater improvement in tocilizumab-treated patients [25]. Based on these data, subcutaneous (but not IV) tocilizumab received FDA approval for SSc-ILD treatment in 2021.

Another post hoc analysis of data from the focuSSced trial assessed the impact of tocilizumab vs. placebo on radiographic ILD progression. Patients with SSc-ILD were categorized by baseline quantitative ILD severity (minimal: < 5%, *n* = 6; mild: > 5–10%, *n* = 25; moderate: > 10–20%, *n* = 54; severe: > 20%, *n* = 48) and quantitative lung fibrosis severity (1st tertile: 0.1–1.0%, *n* = 45; 2nd tertile: 1.1–2.7%, *n* = 44; 3rd tertile: 2.8–18.5%, *n* = 44). Although not powered to assess radiographic differences, the quantitative ILD score improved in the tocilizumab group but worsened in the placebo group at 48 weeks [26].

Rituximab is often used after first-line SSc-ILD therapy failure or in rapidly progressive disease based on the results of open-label studies [22, 27]. While the benefit must be weighed against its association with severe COVID infection [28], data suggest that COVID vaccines can mitigate the risk [29]. The DESIRES trial randomized 56 patients with SSc (mRSS ≥ 10) at four Japanese centers to RTX (375 mg/m^2^ IV weekly for 4 weeks) vs. placebo to assess the impact of RTX on skin disease. In an exploratory analysis of the subgroup of patients with SSc-ILD at baseline (25 of 28 in the RTX group and 23 of 26 in the placebo group), FVC at 24 weeks had slightly improved in the RTX group (+ 0.09%) compared with the placebo group (− 2.9%) suggesting possible benefit [30]. Recently published results of the RECITAL trial conducted at 11 UK centers also support a role for RTX in SSc-ILD.

## Rheumatoid arthritis-associated interstitial lung disease

Interstitial lung disease is a frequent manifestation of rheumatoid arthritis (RA) with significant variation in prevalence (1% to 73%) depending on the screening modality (X-ray, HRCT, PFTs) and population chosen (asymptomatic vs. symptomatic) [5]. Rheumatoid arthritis is different from other CTD-ILDs as the predominant radiologic pattern is usual interstitial pneumonia (UIP) instead of non-specific interstitial pneumonia (NSIP). UIP is characterized by peripheral lower lobe predominant fibrosis with honeycombing and minimal or absent ground glass opacities [31]. The RA-ILD treatment approach is based on expert opinion using observational studies. Glucocorticoids are usually the initial therapy despite the lack of data supporting their use, but most patients will require steroid-sparing agents. MMF is frequently used based on extrapolation of evidence from patients with SSc [14]. However, MMF is not very effective for joint disease, and thus other therapies have been studied. Methotrexate (MTX, dihydrofolate reductase inhibitor), an effective medication for RA joint disease, is often avoided due to its association with lung toxicity [5]. However, recent literature has placed this association in doubt and even a protective effect has been reported [32]. Out of an abundance of caution, bDMARDs (*e.g.*, RTX, abatacept) are often prescribed instead of MTX to control joint disease and abrogate ILD. The initial evidence of RTX benefit in RA-ILD patients was based on retrospective data showing functional and radiographic stabilization or improvement but without assessment of symptomatic response [33]. Two retrospective multicenter studies involving a total of 100 patients showed lung function improvement or stabilization in most patients treated with abatacept [34, 35].

## Idiopathic inflammatory myositis-associated interstitial lung disease

Idiopathic inflammatory myopathies (IIM) are characterized by skeletal muscle inflammation and varying degrees of multiorgan involvement. ILD is frequently seen in patients with polymyositis and dermatomyositis, with a reported prevalence of up to 65 to 78% in some case series using a definition of abnormal imaging or restriction on PFTs [4]. Clinical manifestations in IIMs are closely associated with specific autoantibodies. For example, anti-synthetase antibodies [*i.e.*, anti-aminoacyl-transfer RNA antibodies: anti-histidyl (Jo-1) is most common] are associated with a phenotype consisting of ILD, inflammatory arthritis, mechanic's hands, and Raynaud phenomenon. ILD without evidence of muscle involvement can be the initial presentation. The most common radiologic pattern is NSIP. Glucocorticoids are the standard initial therapy due to their known benefit in the treatment of myositis. A second immunosuppressive medication for treatment, and as a steroid-sparing agent, is often prescribed. Multiple regimens have been used based on experience and mostly observational data. Azathioprine or MMF are usual options for patients with a chronic presentation or mild to moderate ILD [36]. A retrospective study of IIM-ILD patients treated with AZA or MMF at a single center showed that both agents were associated with improved lung function and were useful steroid-sparing agents. The group treated with AZA received lower GC doses than the MMF group, but at the expense of a higher rate of adverse events (AZA 33% vs. MMF 14%) and discontinuation (AZA 17% vs. MMF 7%) [37]. High-dose GC, in addition to CYC, calcineurin inhibitors, or RTX, are options for severe or rapidly progressive IIM-ILD. A subgroup of IIM-ILD patients has positive anti-melanoma differentiation factor 5 antibody (MDA5), associated with rapidly progressive ILD and poor prognosis. A case series of 18 MDA5 + IIM-ILD patients treated with tofacitinib [inhibitor of Janus kinases (JAK)] showed improved survival compared to historical controls (100% vs. 78%). This supports the use of tofacitinib in this specific subgroup of ILD patients [38]. Forty-four IIM-ILD patients were included in the RECITAL trial discussed previously.

## Antifibrotic therapy

Antifibrotic therapy, initially approved for IPF, has also been studied in CTD-ILD. Nintedanib (a tyrosine kinase inhibitor) blocks the activation of multiple downstream profibrotic pathways [39]. Nintedanib was approved for use in patients with IPF after two replicate phase 3 RCTs (INPULSIS-1 and INPULSIS-2) showed it slowed disease progression by reducing FVC decline over 52 weeks. The most common side effect was diarrhea, which was reported in 62.4% of patients, but led to treatment discontinuation in < 5% of patients [10]. In real-world studies, the proportion of patients with IPF who discontinued nintedanib due to any cause has been reported as ranging from 4 to 53%, with differences in study methodologies, patient populations and durations of follow-up possible explanations for this wide range [40].

The role of nintedanib in patients with CTD-ILD was investigated in two subsequent studies. An RCT of nintedanib in SSc-ILD (SENSCIS) included SSc patients with an onset of the first non-Raynaud symptom within the past 7 years and fibrosis affecting ≥ 10% of the lungs. Patients were required to have an FVC > 40% of predicted and a diffusion lung for carbon monoxide (DL_CO_)of 30 to 89% of predicted. Immunosuppressive therapy with prednisone up to 10 mg daily and with MMF or MTX at stable doses for ≥ 6 months was allowed. The primary endpoint was the annual rate of FVC decline (mL/year) assessed over 52 weeks. The difference in the rate of FVC decline between patients receiving active drug versus placebo was 41 ml/year (95% CI: 2.9, 79.0). There was no difference in patient-reported outcomes instruments (SGRQ, HAQ-DI, FACIT-Dyspnea) or skin scores. Diarrhea occurred in 76% of patients and was the most common side effect [15]. Over 52 weeks, adverse events led to permanent discontinuation of treatment in 16% of patients in the nintedanib group and 9% of patients in the placebo group [41]. The relative treatment effect of nintedanib was similar between patients taking and not taking MMF [42]. Data from the whole SENSCIS trial demonstrated a sustained benefit of nintedanib on slowing the progression of FVC decline up to 100 weeks [43]. A subsequent open-label extension study (SENSCIS-ON) showed a similar change in FVC and safety profile after an additional 52 weeks of treatment with nintedanib [44].

The INBUILD trial was a phase 3 RCT that enrolled patients with progressive fibrosing ILD (PF-ILD) [45]. Patients met at least one of the following criteria for progression within the last 24 months: relative FVC decline ≥ 10% predicted; relative FVC decline ≥ 5%– < 10% predicted and worsened respiratory symptoms; relative FVC decline ≥ 5%– < 10% predicted and increased extent of fibrosis on imaging; worsening symptoms and increased extent of fibrosis on imaging. This study included 25.6% (170 of 663) patients with CTD-ILD. It is important to note that AZA, cyclosporine, MMF, tacrolimus, RTX, CYC, or oral GC at a dose of > 20 mg/day were not allowed at randomization. A reduction in FVC decline over 52 weeks, comparable to that seen in IPF studies, and a similar side effect profile were observed. Health-related quality of life evaluated by the KBILD questionnaire was not significantly different between groups.

Pirfenidone (anti-fibrotic and anti-inflammatory agent with activity against IL-1β, IL-6, TNF-α and PDGF) was evaluated in three phase 3 RCTs in IPF and shown to reduce disease progression [11, 46]. A subsequent multicenter phase 2b RCT (RELIEF) examined pirfenidone in PF-ILD other than IPF. The study was terminated early due to futility after slow recruitment and an interim analysis was conducted [47]. A phase II randomized, open-label study (LOTUSS) recruited 63 patients with SSc-ILD (background therapy with prednisone in 17.5% and MMF in 63.5% of patients) who were randomized to receive pirfenidone following a 2-week or 4-week dose escalation schedule to a 2403 mg/day targeted dose. Treatment-emergent adverse events were more common in patients receiving the more rapid dose escalation and most commonly included headache and fatigue. Concurrent MMF did not impact adverse events. There was no significant difference in patient-reported outcomes as evaluated by the Mahler TDI and HAQ-DI [48]. These data paved the way towards Scleroderma Lung Study III, for which the analysis is underway.

Pirfenidone was also studied in RA-ILD in the TRAIL1 trial. This study randomized patients with a diagnosis of RA based on the 2010 ACR-EULAR criteria and an HRCT scan showing fibrosis affecting > 10% of the lung. Patients were required to have an FVC > 40% of predicted, DL_CO_ > 30% of predicted, and < 10% relative change in FVC between screening visit and baseline. The exclusion criteria included introduction or dose modification of any immunosuppressive therapies to manage pulmonary manifestations of RA within 3 months of screening. The primary outcome was a composite endpoint of decline in FVC predicted of 10% or more or death within the 52-week trial period. There was no significant difference in dyspnea between groups as evaluated by the Dyspnea-12 score. The study was stopped early because of slow enrollment (123 of a target of 270 patients). There was no difference between groups in the primary outcome (11% in the pirfenidone group vs 15% in the placebo group, OR 0·67 [95% CI 0·22 to 2·03]; *p* = 0·48) [49].

## Hematopoietic stem cell transplant

Autologous stem cell transplant (ASCT) is a non-curative therapy (most patients remain on immunosuppression following the intervention) for a select group of patients with severe or refractory SSc. Referral to experienced ASCT centers is recommended because treatment-related morbidity and increased short-term mortality have been described. Lymphoablative and myeloablative regimens have shown benefit (Table 4) [50–52].

**Table 4:** Autologous stem cell transplant studies in patients with systemic sclerosis

Trial	ASSIST [52] (2011)	ASTIS [51] (2014)	SCOT [50] (2018)	Del Papa et. al. [53] (2017)	NISSC1 [54] (2021)
Biological effect of ASCT	Non-myeloablative	Lymphoablative	Myeloablative	Unspecified	Non- myeloablative
Design	Single center, prospective, randomized	Multicenter, prospective, randomized	Multicenter, prospective, randomized	Single center, retrospective, observational	Multicenter, prospective, observational
Inclusion criteria	Age < 60, cutaneous involvement proximal to elbow or knee with mRSS > 14, internal organ involvement (DL_CO_ < 80%, decrease in FVC by ≥ 10% in 12 months, pulmonary fibrosis, abnormal ECG, or GI tract involvement)	Age 18–65, diffuse cutaneous SSc for < 4 years, mRSS > 14, cardiac, pulmonary, or renal involvement	Age 18–69 with SSc for ≤ 5 years with pulmonary or renal involvement	SSc for < 4 years, mRSS ≥ 14, European Scleroderma Study Group (ESSG) clinical activity score ≥ 3	Age 18–65, established SSc, autologous HSCT
ASCT participants	10	79	36	18	80
Control participants	9	77	39	36	NA
Total body irradiation	No	No	Yes	No	No
CD34 + cell mobilization	CYC 2 g/m^2^ IV × 1 d plus G-CSF 10 µg/kg SC from day 5 post CYC until apheresis	CYC 4 g/m^2^ IV ~ 100 mg/Kg for 2 days G-CSF 10 µg/Kg/day	G-CSF 16 µg/kg/day for 4 days	CYC 4 g/m^2^ for 2 days and G-CSF 10 µg/kg	CYC 1–4 g/m^2^ and G-CSF, dose unspecified
Conditioning regimen	CYC 200 mg/Kg IV plus mesna day − 5 to day − 2, ATG 0.5 mg/Kg IV day − 5, 1.5 mg/Kg day − 4 to day − 1 plus GC 1000 mg	CYC 200 mg/Kg IV for 4 days ATG 7.5 mg/Kg for 3 days GC 1 mg/Kg	CYC 120 mg/Kg IV plus mesna for days − 3 to − 2 and ATG 90 mg/Kg on days − 5, − 3, − 1, + 1, + 3, + 5	CYC 200 mg/kg IV with mesna day − 5 to − 2 and ATG 7.5 mg/kg with GC IV 1 mg/kg day − 3 to − 1	CYC 200 mg/kg IV (4 patients received thiotepa 10 mg/kg and CYC 100 mg/kg) ATG (varied dosing) ± GC at unspecified dose
Controls	1.0 g/m^2^ IV CYC plus mesna monthly for 6 mo	750 mg/m^2^ IV CYC monthly for 12 mo	500 mg/m^2^ IV CYC at baseline followed by 750 mg/m^2^ IV CYC and mesna for 11 mo	1 g IV CYC monthly for ≥ 6 mo. plus 5–10 mg prednisone or MTX (10–20 mg w.) or AZA (100–200 mg/day), plus low-dose prednisone (5–10 mg/day), or pulse methylprednisolone followed by low-dose AZA, unspecified dose	Not applicable
Primary outcome	mRSS decrease or FVC increase at 12 months after treatment randomization	Event-free survival, defined as the time in days from randomization until the occurrence of death due to any cause or development of persistent major organ failure—heart, lung, or kidney	Global rank composite score (ranking system that accounts for death, failure of event free survival, FVC, HAQ-DI, and mRSS) at 54 months	mRSS, DLco, and disease activity, according to European Scleroderma Study Group scoring system (ESSG)	Progression-free survival defined as survival after ASCT without death or progression of SSc
Follow-up	5 y	median 5.8 y	Up to 6 y	Up to 5 y	Median 2 y
Results	All patients in HSCT group improved at, or before, 12 mo	Increased treatment-related mortality in first year, but long-term event-free survival benefit	Superiority in transplant group	Higher survival rate, reduced skin involvement and disease activity, and preservation of lung diffusing capacity in treatment group	Progression- free survival rate of 81.8% at 2 y

*ASSIST* autologous non-myeloablative haemopoietic stem-cell transplantation compared with pulse cyclophosphamide once per month for systemic sclerosis, *ASTIS* autologous stem cell transplantation international scleroderma, *SCOT* scleroderma, cyclophosphamide or transplantation, *NISSC1* autologous stem cell transplantation for progressive systemic sclerosis: a prospective non-interventional study from the European society for blood and marrow transplantation autoimmune disease working party, *CYC* cyclophosphamide, *G-CSF* granulocyte colony stimulating factor, *ATG* anti-thymocyte globulin, *GC* glucocorticoid, *mRSS* modified Rodnan skin score, *MTX* methotrexate, *AZA* azathioprine, *y* years, *d* days, *mo* months, *SC* subcutaneous, *FVC* forced vital capacity % predicted

## Lung transplant

Lung transplant is a viable option for some patients who develop PF-ILD despite maximal therapy. It is recognized that the extrapulmonary manifestations of CTD can affect transplant outcomes. There is significant concern about esophageal dysmotility and reflux in SSc patients. Other extrapulmonary comorbidities, such as muscle weakness, osteoporosis, and cardiac, central nervous system, or renal involvement, can impact post-transplant outcomes and can be contraindications depending on severity. Patients with CTD may have an increased risk of venous thromboembolism and allosensitization [55]. Despite these issues, studies have shown that post-transplant outcomes are similar in CTD-ILD compared to other indications [56]. The most recent consensus guidelines emphasize early referral to allow modifiable risk factor identification that can impact transplant candidacy or post-transplant outcomes [57].

## Conclusion

There are a growing number of clinical trials testing the efficacy of drugs that target various pro-inflammatory and pro-fibrotic pathways in patients with CTD-ILDs. As our understanding of disease pathogenesis increases, undoubtedly there will be additional agents developed that warrant testing. The need to conduct well-designed clinical trials that consider the natural history of disease will be of paramount importance. Based on CTD-ILD trials conducted to date, there is a growing therapeutic armamentarium including anti-inflammatory agents (e.g., MMF, CYC, tocilizumab and RTX) and procedures (*e.g*., autologous stem cell transplant), anti-fibrotic agents (*e.g.*, pirfenidone and nintedanib), and combination therapy. The most common outcome measure in ILD trials is FVC % predicted, but secondary analyses making use of computer-aided quantification of lung disease can provide additional insights. Standardizing study outcomes including patient-reported outcome instruments used in CTD-ILD clinical trials would be useful to permit between-study comparisons. Given the growing body of clinical trial data to inform the treatment of patients with CTD-ILD, ongoing efforts by the international community of rheumatologists, pulmonologists, radiologists, medical imaging experts, and patients to publish updated treatment guidelines are timely.

## Data Availability

Not applicable.

## Abbreviations

ILD: Interstitial lung disease
CTD: Connective tissue disease
HRCT: Chest high-resolution computed tomography
IPF: Idiopathic pulmonary fibrosis
PFT: Pulmonary function test
RCT: Randomized-controlled trial
RTX: Rituximab
CYC: Cyclophosphamide
FVC: Forced vital capacity
SD: Standard deviation
DLCO: Diffusion capacity for carbon monoxide
PRO: Patient-reported outcomes
SGRQ: St. George's Respiratory Questionnaire
KBILD: King's Brief Interstitial Lung Disease
IV: Intravenous
EQ-5D: European Quality of Life Five-Dimension
GC: Glucocorticoids
VAS: Visual analog scale
SLSI: Scleroderma Lung Study I
SF-36: Short-Form 36
HAQ-DI: Health assessment questionnaire-disability index
SHAQ-DI: Scleroderma health assessment questionnaire-disability index
SLSII: Scleroderma Lung Study II
MMF: Mycophenolate mofetil
UCLA: University of California Los Angeles3
FAST: Fibrosing Alveolitis in Scleroderma Trial
AZA: Azathioprine
EULAR: European League Against Rheumatism
FDA: US FOod
bDMARD: Biologic disease-modifying anti-rheumatic drug
CRP: C-reactive protein
ESR: Erythrocyte sedimentation rate
FACIT: Functional assessment of chronic illness therapy
UK: United Kingdom
RA: Rheumatoid arthritis
UIP: Usual interstitial pneumonia
NSIP: Non-specific interstitial pneumonia
MTX: Methotrexate
IIM: Immune-medicated inflammatory myositis
MDA-5: Melanin Differentiation Factor-5
JAK: Janus-kinase
ASCT: Autologous stem cell transplantation
