# Correction: Learnings from clinical trials in patients with connective tissue disease‑associated interstitial lung disease

**DOI:** 10.1186/s13075-023-03123-6

**Published:** 2023-07-27

**Authors:** Jean Paul Higuero Sevilla, Areeka Memon, Monique Hinchcliff

**Affiliations:** 1 Department of Internal Medicine, Section of Pulmonary, Critical Care and Sleep Medicine, Yale School of Medicine, New Haven, CT 06520 USA; 2 Department of Internal Medicine, University of Connecticut School of Medicine, Farmington, CT 06032 USA; 3 Department of Internal Medicine, Section of Rheumatology, Allergy & Immunology, Yale School of Medicine, 300 Cedar Street, The Anlyan Center PO BOX 208031, New Haven, CT 06520 USA

**Correction: Arthritis Res Ther 25, 118 (2023)**

**https://doi.org/10.1186/s13075-023-03090-y**

Following publication of the original article [1], the authors identified an error to Jean Paul Higuero Sevilla’s last name. The correct last name should be “Higuero Sevilla” instead of “Sevilla”.

The author group has been updated above and the original article [1] has been corrected.
